# Standing Long Jump Performance Is Enhanced When Using an External as Well as Holistic Focus of Attention: A Kinematic Study

**DOI:** 10.3390/s24175602

**Published:** 2024-08-29

**Authors:** Esmaeel Saemi, Alireza Hasanvand, Mohammadreza Doustan, Ayoub Asadi, Kevin Becker

**Affiliations:** 1 Department of Motor Behavior and Sport Psychology, Faculty of Sport Sciences, Shahid Chamran University of Ahvaz, Ahvaz 6135783151, Iran; a.hasanvand98@gmail.com (A.H.); m.doustan@scu.ac.ir (M.D.); 2 Department of Kinesiology, Iowa State University, Ames, IA 50011, USA; asadi68@iastate.edu; 3 Department of Kinesiology, Recreation, and Sport Studies, University of Tennessee, Knoxville, TN 37996, USA; kbecker2@utk.edu

**Keywords:** internal focus of attention, external focus of attention, holistic focus of attention, maximum knee flexion angle

## Abstract

Standing long jump is known as one of the important skills in the success of athletes in most sports. In addition, one of the most effective factors that can affect standing long jump distance and kinematics is the focus of attention used by the athlete. Therefore, the aim of the present study was to compare the effect of internal, external, and holistic focus of attention instructions on standing long jump performance and kinematics. The participants were 30 novices (all males; mean age = 21.70 ± 2.21 years; mean height = 175.73 ± 6.09 cm; and mean weight = 73.76 ± 11.77 kg) who performed 12 standing long jumps in four focus of attention conditions. Internal focus, external focus, holistic focus, and control conditions were implemented in a counterbalanced order. Jump distance and maximum knee flexion angle before take-off were recorded in all trials. The results showed that in relation to the standing long jump performance, the distance was similar in external and holistic focus conditions, and both were superior to internal or control conditions. There was no difference between control and internal focus of attention conditions. The results related to movement kinematics, however, did not report a difference between the maximum flexion angles before take-off. This study replicates the benefits of external and holistic focus instructions for jump distance, but this difference was not a product of different maximum knee flexion angles. It is suggested that coaches implement external and/or holistic focus cues to maximize athlete performance in jumping tasks.

## 1. Introduction

The standing long jump is known as an important skill in the success of athletes in most sports that require full coordination of the whole body, such as basketball, parkour, etc. [1,2,3]. This task is sometimes even used in physical education classes to measure one dimension of the physical fitness of students [4], so considering the importance of this task, the best instructions and technique should be used in its implementation [1,3,4,5].

According to several studies, the focus of attention an athlete chooses when jumping is an important factor that can affect jump distance and the underlying kinematics that influence distance [1,6,7,8]. The focus of attention has typically been considered in a dichotomy, comparing an internal and external focus of attention, which has been studied by researchers in different skills for over 25 years [9,10,11]. Based on the extant evidence in this body of literature, it appears that the use of an external focus of attention during the performance of sports skills, regardless of the level and type of skill, can be superior to the use of an internal focus of attention on motor performance and learning [11]. For example, and specifically in the standing long jump task, Porter et al. [12] reported that the use of verbal instructions, like external focus of attention, compared to internal focus of attention or a condition without attentional instructions, can lead to an improvement in standing long jump performance in novice performers. These findings were repeated by other researchers such as Becker et al. [13] and Noroozi et al. [14]. However, some studies could not report the superiority of using an external focus of attention in novice [15] or skilled performers [16]; therefore, considering the importance of this research topic, conducting more studies is needed in this context.

Internal focus of attention is defined as focusing on limb movements while performing a motor skill (e.g., focusing on knee joint extension during a standing long jump), while an external focus of attention focuses on the effects of movement in the environment (e.g., focusing on jumping toward a cone placed in front of you; Noroozi et al. [12,14]). One of the perspectives that justify the superiority of external focus of attention over internal focus of attention is the constrained action hypothesis [17]. According to this hypothesis, when using an internal focus of attention, the learner’s motor system is involved in conscious processing, and therefore disrupts movements that may be capable of more automatic organization. On the contrary, when adopting an external focus of attention, automatic control processes of movement are facilitated by planning a movement at the level of an outcome, and therefore motor learning and performance are improved [17,18]. In addition, recently, Wulf and Lewthwaite [19], in the OPTIMAL theory of motor learning, focused on the positive effects of external focus of attention along with other motivational variables such as self-controlled practice [20,21] and enhanced expectancies [22,23]. These authors emphasized that external focus of attention can facilitate motor learning and performance, along with other motivational variables, by facilitating the coupling of goal and action [19].

In addition to the external and internal foci of attention that have been compared for 25 years in different studies and in different skills [11], recently, another type of focus of attention instruction has been introduced in the literature. A holistic focus of attention draws attention to the general feeling or sensations associated with completing a skill (e.g., feeling explosive in a standing long jump; Becker et al. [13]). This type of focus cue has been found to benefit motor performance relative to internal focus in standing long jumps [13,14,24], underhand shot throws [25], and isokinetic elbow flexions [26]. In most cases, performance when using an external or a holistic focus does not differ, but both show a benefit over an internal focus. Given the perceived similarities in these benefits, it is important to understand if they share similar mechanisms underlying the performance benefits. Despite the recent support for the superiority of holistic focus of attention [14,24,26], some studies have not been able to demonstrate the superiority of holistic over internal focus of attention in skilled or novice performers [15,27]; therefore, more studies are still needed in this field to determine the generalizability of, and underlying contributors to, the benefits of holistic focus.

One means of investigating the underlying mechanisms of performance improvement in the standing long jump task is exploring movement kinematics [28,29,30]. It has been found that there is a positive relationship between some kinematic variables, such as the maximum knee flexion angle and jump distance, during the long jump [1,6,7,8]. In this context, some researchers have reported that using an external focus of attention compared to an internal focus of attention and conditions without attention instructions can increase long jump performance while also increasing the maximum knee flexion during jumping in skilled and novice performers [1,6,7,8]. Due to the novelty of the holistic focus of attention instruction in the field of motor performance, the majority of studies have reported only behavioral outcomes without kinematic analyses. According to the knowledge of the present researchers, so far, no study has investigated and compared changes in maximum knee flexion angle before take-off in the standing long jump task in the holistic focus of attention compared to the internal, external, and control conditions. Given that an external or holistic focus consistently improves standing long jump distance relative to an internal focus, the aim of the present study was to determine if these changes can be partially explained by differences in maximum knee flexion angle before take-off in different focus conditions. Considering the similarity of the effect of the holistic focus of attention to the external focus of attention [14,24,26], in this study, it was predicted that standing long jump performance would be similar when using a holistic focus of attention and external focus of attention, and both would be superior to internal focus of attention and control conditions. It was also predicted that the maximum knee flexion angle before take-off would be higher in external and holistic attention conditions than in internal as well as control conditions.

## 2. Materials and Methods

### 2.1. Participants

The sample size was 30 participants. This was calculated by GPower (version 3.1.9) and based on indicators such as significance level = 0.05, statistical power = 0.90, and effect size = 0.25 [24]. The ANOVA statistical test was used with repeated measurements. Therefore, 30 undergraduate students (all males; mean age = 21.70 ± 2.21 years; mean height = 175.73 ± 6.09 cm; and mean weight = 73.76 ± 11.77 kg) participated in this study with a within-participant design. The inclusion criteria of the study were: (1) being in good physical health and not having any joint or muscle injuries; and (2) being between 18 and 30 years old. Exclusion criteria included: (1) unwillingness to cooperate at any time during the research; and (2) occurrence of any physical injury that could impact the experiment. The research plan was prepared based on the Declaration of Helsinki and was also approved by the research committee of the Department of Motor Behavior and Sport Psychology of the University.

### 2.2. Task and Apparatus

#### 2.2.1. Performance Variable

Performance in the present study was recorded as standing long jump distance (cm). The standing long jump task has been used in various studies [1,14]. In this research, a scaled rubber floor with a width of 1 m and a length of 4.5 m was used to complete the jumping task. The starting point was marked by a white line on the flooring. After each standing long jump, the distance between the starting line and the back of the jumper’s heel was recorded by a research assistant with the use of a tape measure.

#### 2.2.2. Kinematic Variable

**Figure 1:**
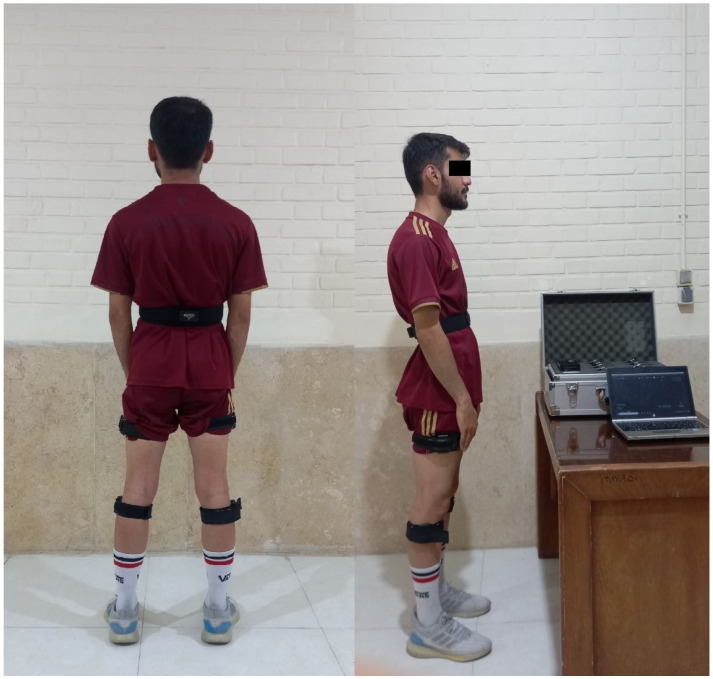
Illustration of sensor placement on human body.

A motion analysis system based on commercial Inertial Measurement Units (IMU) sensors (IMU Motion Capture Setup V2.0.7; BSN 1-15, software: B2.0.1.0) was used to measure the kinematic variables related to the participants’ knee joints. In other words, to measure the maximum knee flexion angle before take-off, during the standing long jump, from one sensor on the torso (on the T8 axis and on the frontal plane as a reference), two sensors on the thigh (in the middle part and in the sagittal plane; both legs), and two sensors on the shin (middle section and in the sagittal plane; both legs) were used (five sensors in total). The sensors were placed on different parts of the body so that the *x*-axis was parallel to the ground and the *y*-axis was perpendicular to the ground (Figure 1). For each jump, data from the five sensors were simultaneously recorded by the B2.0.1.0 software installed on the laptop. After the end of all trials (12 jumps), the average maximum knee flexion for each condition (three trials for each condition) was calculated for both legs and extracted as an output in an Excel file. This device has been used in previous studies to measure kinematic variables [31]. Maximum knee flexion angle was defined as the largest knee flexion angle observed before take-off [1,7,8,32].

### 2.3. Procedures

After selecting the participants for the study, the participants were asked to go to the lab to participate in the experiment individually. During the experiment, only the participant and two examiners were present in the lab. The participants were not aware of the purpose of the study and only received basic instructions on how to correctly perform the standing long jump task. At the beginning and after initial explanations were given to the participants, all participants warmed up for 5 min. The warm-up consisted of some stretching movements at the beginning, followed by some jumping movements. Next, five sensors were placed on the participant’s body to record kinematic variables. Then, the participant stood behind the starting line and started jumping when instructed. Each participant performed 12 jumps in four blocks of three trials. Between each block of three trials, each participant had a 2 min rest, and 30 s rest periods were taken between each trial. After each trial, the jump distance of the participants was recorded in centimeters as the distance between the starting point and the heel of the foot landing on the floor. The participants performed 12 jumps in such a way that all three trials were in the same focus of attention and control condition. Their first three jumps were performed in a control condition, meaning they received no attentional instructions and were only asked to try to perform their best jump. After that, the participants jumped in three internal, external, and holistic focus of attention conditions (three trials in each condition) in a counterbalanced order. In the internal focus of attention, participants received this instruction; “As you try to perform your best jump, try to mentally focus on extending your knee joint during the jump”. In the external focus of attention, the instruction was: “As you try to perform your best jump, try to mentally focus on a cone that was located in front of you at a distance of 5 m from the starting line”. In the holistic focus of attention, the instruction was “As you try to perform your best jump, try to mentally focus on the smoothness and fluidity of the entire jump movement”. These instructions were taken from previous related studies [14]. After each condition of internal, external, and holistic focus of attention, all participants answer two questions: (1) In this trial, did you focus your attention according to the received instructions?; and (2) What percentage of time did you focus your attention according to the received instructions?

### 2.4. Data Analysis

To analyze the data from this study, first, descriptive statistics were used to report the average and standard deviation of demographic data as well as research variables. Separate ANOVAs with repeated measures (4) were used to compare three attentional and control conditions according to the performance (long jump distance) and kinematic (maximum knee flexion angle before take-off) variables. Preliminary investigations showed that all the assumptions required for using inferential statistics like repeated measure ANOVA were met. The Bonferroni follow-up test was used for pairwise comparisons. For each attention condition, the average scores for all three trials were calculated and analyzed. A significance level of 0.05 was considered for this study and SPSS software (version 24) was used for data analysis. The intra-class correlation (ICC) was calculated for both dependent variables. The ICC for the long jump distance was 0.913, and for the maximum knee flexion angle, it was 0.912, which shows that both instruments had good reliability.

## 3. Results

### 3.1. Jump Distance

**Figure 2:**
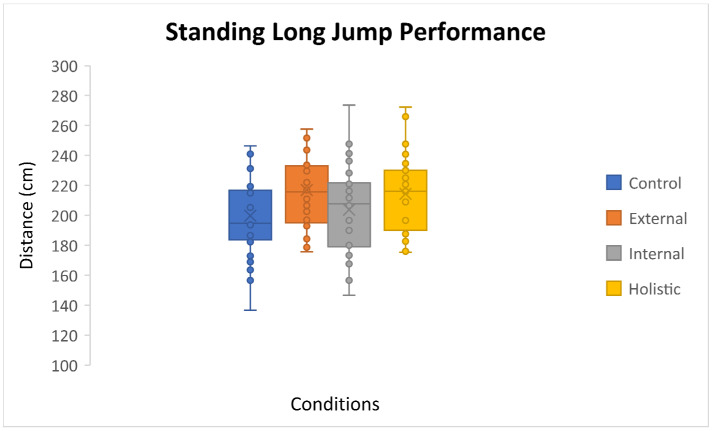
Boxplot of standing long jump performance for all attentional conditions.

The assumption of the Mauchly’s test of sphericity was not violated for jump distance. The results of the one-way ANOVA with repeated measures showed that there was a significant difference between the different attentional instructions; F(3,87) = 16.68, *p* = 0.0001, partial η^2^ = 0.36. The results of the Bonferroni follow-up test showed that the long jump performance in the external (216.92 ± 27.73) and holistic focus of attention (214.41 ± 25.51) conditions were similar (*p* > 0.05), and both conditions were superior to internal focus of attention (203.82 ± 29.64; respectively, *p* = 0.0001 and *p* = 0.002) and control conditions (199.81 ± 29.16; respectively, *p* = 0.0001 and *p* = 0.001). There was no difference between the internal focus of attention and control conditions (*p* > 0.05; Figure 2).

### 3.2. Maximum Knee Flexion Angle

**Figure 3:**
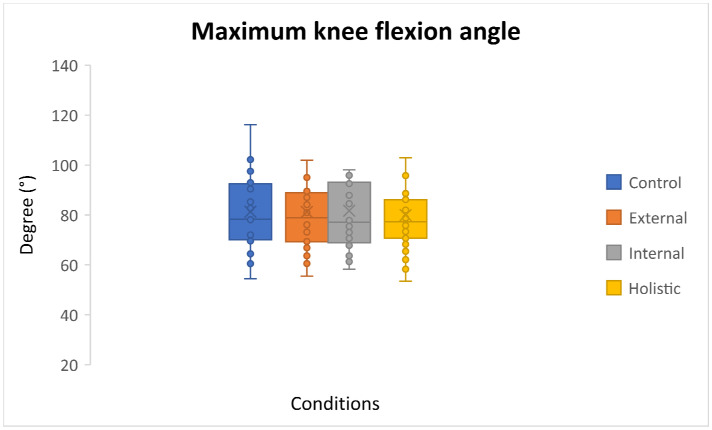
Boxplot of the maximum knee flexion angle for all attentional conditions.

The assumption of the Mauchly’s test of sphericity was not violated for maximum knee flexion angle. The results of the one-way ANOVA with repeated measures showed that there was no significant difference between the different attentional instructions: F(3,87) = 0.67, *p* = 0.57, partial η^2^ = 0.02. In other words, the average angles of maximum knee flexion for all three instructional conditions of internal (81.69 ± 18.68), external (81.20 ± 16.67), and holistic (79.94 ± 15.93), along with the control conditions (81.30 ± 18.11), were similar (Figure 3).

### 3.3. Compliance Check

The findings related to the attention instruction compliance checklist showed that all participants responded positively to the first question (that is, all said that they acted according to the requested attentional instructions). For the second question, adherence scores were similar in the internal focus of attention (80.91%), in the external focus of attention (82.40%), and in the holistic focus of attention (86.46%), indicating participants had acted according to the requested instructions.

## 4. Discussion

The aim of the present study was to compare the effects of internal focus, external focus, holistic focus, and no attentional instruction (control) conditions in performing the standing long jump skill by considering distance and a kinematic outcome. The results of this study showed that the standing long jump performance in external and holistic focus conditions was similar, and both were superior to the internal focus and control conditions. However, no difference was observed in the maximum knee flexion angle before take-off between different attentional conditions. In addition, due to the high percentage of compliance with the attentional instructions reported by the participants in all conditions (all were above 80%), it can be reasonably concluded that the observed difference between the attentional conditions happened precisely because of the attentional manipulation.

The performance (distance) results of this study are in line with several previous attentional focus studies utilizing jumping skills [1,8,12,13,14,24]. Most of these studies, similar to the findings of the present study, showed that an external focus of attention is superior to an internal focus of attention and can improve jumping performance. Some other research has also reported that external and holistic focus of attention are similar and both superior to internal focus of attention and can improve long jump performance in skilled and novice individuals [13,14,24]. For example, Noroozi et al. [14] observed that skilled and novice female karateka performed similarly in external and holistic focus of attention conditions, which were superior to internal focus of attention and control conditions. These findings were also reported by Becker et al. [13] as well as Zhuravleva et al. [24]. Importantly, though the skill was the same in the above-mentioned studies (standing long jump), the present study shows novelty in using a different holistic focus cue (explosive vs. smoothness and fluidity). This is an important finding as it shows that focusing on different types of feelings and sensations that occur during a successful jump both demonstrate a benefit. Thus, it is not simply one cue (feeling explosive) that supports the benefit of a holistic focus. Studies using other motor tasks have not always found a benefit of an external and holistic focus [15,16,27,33], but those studies using jumping tasks have consistently found these strategies to be effective at enhancing jump distance or height.

Our performance differences between an internal and external focus are consistent with the predictions of both the constrained action hypothesis and the attentional branch of the OPTIMAL theory [17,19]. These perspectives suggest that an external focus enhances performance by promoting automaticity or greater goal–action coupling by focusing attention on the action effect (external focus) instead of the action (internal focus). However, they are limited by considering an external focus as the only possible strategy to create this benefit. The benefits of focusing on action effects were emphasized in common coding theory, which provided the theoretical underpinnings of the constrained action hypothesis [34]. It is important to note that an external focus as defined in the literature is only one type of action effect that may be used. A holistic focus (e.g., focusing on feeling smooth or explosive) directs attention to an action effect occurring within the body, which has previously been described as “close effects” [10] or resident effects [35]. Our evidence here, along with a number of previous studies, supports the notion that both a holistic and external focus allow performers to connect with different types of action effects, which promotes superior performance relative to focusing on the action itself with an internal focus.

Although the findings of the present study reported the superiority of an external and holistic focus of attention compared to an internal focus of attention and control condition, the kinematic findings related to the maximum knee flexion angle were similar in all conditions and a difference was not found between different attentional instructions. Previous studies have provided evidence for an external focus resulting in increased maximal knee flexion prior to take-off relative to an internal focus, while also leading to an increase in jump distance or height [1,7,8].

In the present study, the number of participants was 30 novices who performed the standing long jump in different attentional focus conditions, and the kinematic variable (maximum knee flexion angle before take-off) was recorded in each condition. Other related studies that have shown a significant difference between different attentional focus conditions in the kinematic variable mostly had a smaller sample size [1,7]. For example, Asadi et al. [1] had 15 participants and Gokeler et al. [7] had 16 participants; perhaps one of the reasons for the different results of the present study is related to the sample size. A larger sample size can reduce the likelihood of detecting a false positive, so it is suggested that the future related studies be conducted with a higher sample size to provide more accurate results. Additionally, Gokeler et al. [7] was related to patients after ACL reconstruction, while in the present study, the participants were healthy adults; perhaps another reason for this inconsistency is related to the difference in the jumping pattern in healthy participants compared to patients after ACL reconstruction. In addition, the type of jump in Gokeler et al. [7] was a single leg hop jump, while the type of jump in the current study was standing long jump; another reason for this difference in the results could be due to the difference in the nature of these two types of jumps. Therefore, it is suggested that some future studies compare both healthy participants and patients after ACL reconstruction in different attentional focus conditions in standing long jump and record different related kinematic variables.

Indeed, we focused on this particular kinematic variable (maximum knee flexion angle before take-off) as it has been previously identified in three separate studies as a potential underlying contributor to differences in jump outcomes and could be easily measured outside of a biomechanics laboratory. However, in this study, jump distance differed among focus conditions without any differences in maximal knee flexion. Since an external and holistic focus presumably allow for more self-organization in the movement solution, it is possible that these conditions led to a solution that produced a mechanical advantage that was not strictly a result of maximal knee flexion. Previous studies have demonstrated that an external focus results in increases in functional variability through the organization and coordination of multiple joints involved in the movement [36]. Using the uncontrolled manifold technique, these authors demonstrated that a performance variable can be enhanced through complex combinations of individual kinematic variables. Thus, one of the limitations of the current study was only measuring one kinematic variable (maximum knee flexion angle before take-off). This was chosen to accomplish the study in a more applied environment using a limited number of inertial movement units as opposed to a full motion capture system, but may have limited our ability to detect differences in kinematics at other joints.

Future studies should consider a more complex kinematic analysis to understand the contributions of differences present at individual joints, as well as using more complex techniques to understand how variability in movement at multiple joints collectively influence jump distance. For example, it is suggested that the future studies measure and investigate the kinematic variables of other body parts, such as the movement of the arms and hands during the standing long jump; perhaps the mechanisms involved in the superiority of external and holistic focus of attention can be found in the differences in the kinematic indices of the movements of the upper body during jumping. Additionally, due to the existence of other possible mechanisms in justifying the superiority of external and holistic focus of attention, such as mechanisms related to kinetic variables (e.g., differences in ground reaction forces), it is suggested that other related studies try to replicate the present study by considering and recording different kinetic variables related to the standing long jump. Certainly, considering the different kinetic and kinematic variables related to the standing long jump in more depth while providing different attentional focus instructions can increase our understanding of the mechanisms involved in the superiority of external and holistic focus of attention over other attentional focus instructions.

## 5. Conclusions

In summary, the findings of the current study replicated the benefit of a holistic focus or external focus in enhancing performance in a jumping task [13,14,24,26]. Importantly, the holistic focus condition used a different cue that connected performers to a relevant but unique feeling associated with the movement (explosive vs. smoothness and fluidity), demonstrating the versatility of a holistic focus. In the present study, we failed to detect a difference in the kinematic variable of maximum knee flexion angle. This was unique compared to previous research comparing an internal and external focus, and suggests that the differences in jump distance presented here are likely a result of other untested kinetic or kinematic differences. Future work should explore this question with a more comprehensive kinematic and/or kinetic analysis. From a practical standpoint, this study adds to the growing evidence that both an external and holistic focus can be useful strategies to improve the jumping performance of athletes. Practitioners working with athletes should use this knowledge to develop a robust body of cues that fit within these definitions to find the most effective cues to use with each athlete.

## Data Availability

The datasets used and/or analyzed during the current study are available from the corresponding author on reasonable request.
